# Recent Advances of PDMS In Vitro Biomodels for Flow Visualizations and Measurements: From Macro to Nanoscale Applications

**DOI:** 10.3390/mi15111317

**Published:** 2024-10-29

**Authors:** Andrews Souza, Glauco Nobrega, Lucas B. Neves, Filipe Barbosa, João Ribeiro, Conrado Ferrera, Rui A. Lima

**Affiliations:** 1 MEtRICs—Mechanical Engineering and Resource Sustainability Center, Mechanical Engineering Department, University of Minho, Campus de Azurém, 4800-058 Guimarães, Portugal; andrewsv81@gmail.com (A.S.); glaucotvn@hotmail.com (G.N.); neves.lucas17@gmail.com (L.B.N.); a88077@alunos.uminho.pt (F.B.); 2 CMEMS-Uminho—Center for Microelectromechanical Systems, Mechanical Engineering Department, University of Minho, Campus de Azurém, 4800-058 Guimarães, Portugal; 3 CIMO—Mountain Research Center, Instituto Politécnico de Bragança, 5300-252 Bragança, Portugal; jribeiro@ipb.pt; 4 Instituto Politécnico de Bragança, 5300-252 Bragança, Portugal; 5 Departamento de Ingeniería Mecánica, Energética y de los Materiales, Universidad de Extremadura, 06006 Badajoz, Spain; cfll@unex.es; 6 Instituto de Computación Científica Avanzada (ICCAEx), Universidad de Extremadura, 06006 Badajoz, Spain; 7 CEFT—Transport Phenomena Research Center, Faculdade de Engenharia da Universidade do Porto (FEUP), Rua Roberto Frias, 4200-465 Porto, Portugal

**Keywords:** polydimethylsiloxane, PDMS applications, in vitro biomodels, microfluidics, blood flow, biomedical engineering

## Abstract

Polydimethylsiloxane (PDMS) has become a popular material in microfluidic and macroscale in vitro models due to its elastomeric properties and versatility. PDMS-based biomodels are widely used in blood flow studies, offering a platform for improving flow models and validating numerical simulations. This review highlights recent advances in bioflow studies conducted using both PDMS microfluidic devices and macroscale biomodels, particularly in replicating physiological environments. PDMS microchannels are used in studies of blood cell deformation under confined conditions, demonstrating the potential to distinguish between healthy and diseased cells. PDMS also plays a critical role in fabricating arterial models from real medical images, including pathological conditions such as aneurysms. Cutting-edge applications, such as nanofluid hemodynamic studies and nanoparticle drug delivery in organ-on-a-chip platforms, represent the latest developments in PDMS research. In addition to these applications, this review critically discusses PDMS properties, fabrication methods, and its expanding role in micro- and nanoscale flow studies.

## 1. Introduction

Polydimethylsiloxane (PDMS) is an elastomeric polymer known for its astonishing properties, including biocompatibility, resistance to biodegradation, chemical stability, gas permeability, good mechanical properties, exceptional optical transparency, and simple fabrication [1]; being capable of replicating submicron features to create microstructures [2] and being possible to change almost all properties easily as permeability with organophilic nano-silica [3]; or changes in mechanical properties variating the cure agent concentration [4]. Furthermore, PDMS has hyperelastic behavior, also seen in biological tissues, and variable elasticity, which is one of its most remarkable advantages; as a result, this elastomer is attracting increasing attention in the biomedical field. As summarized in Table 1, PDMS’s most relevant properties make it ideal for microfluidic devices and in vitro biomodels.

These unique qualities have led to the widespread use of PDMS in a variety of applications, including micropumps [14], microvalves [11], optical systems [15,16], in vitro blood studies [17,18], blood analogs [19], implants [20,21], and microfluidics [22,23]. PDMS is the most frequently used material to fabricate biomedical microdevices, crucial for developing systems like drug delivery, clinical diagnostics, and point-of-care testing [24]. The materials employed in these systems should be optically transparent and biocompatible, allow for fast prototyping, and be low-cost [25], which are present in PDMS.

Besides its applications in biomicrofluidics, PDMS has been extensively used in the creation of in vitro biomodels to investigate blood flow and related phenomena in diseases, including aneurysms and stenosis [26,27,28,29,30,31]. The PDMS biomodels have an exceptional replication of the artery lumen and exceptional optical transparency, making them an ideal choice for the application of optical techniques like particle tracking velocimetry (PTV), particle image velocimetry (PIV), and confocal micro-PIV [32,33,34,35]. These experimental flow studies have improved our understanding of cardiovascular diseases, validated numerical methods, and examined the performance of stents and other medical devices [36,37,38].

Glass and polymers such as PDMS and polymethylmethacrylate (PMMA) are the most frequently used materials to fabricate microfluidic devices and biomodels due to their remarkable optical transparency. However, PDMS, due to its unique mechanical properties, has become the most used material to produce devices [39,40,41]. Table 2 shows the main advantages and disadvantages of PDMS, PMMA, 3D printing resins, and glass.

Despite the advantages of PDMS shown in Table 2, this polymer has some limitations. PDMS presents a hydrophobic surface [12,47,48], which can limit its application in some biological samples [49]. The hydrophobicity of PDMS in microchannels increases the flow resistance and makes it complex to wet the surface of the channels with liquids. In addition, PDMS tends to swell [6,23] when combined with specific chemicals. Moreover, it is challenging to perform quantitative analysis of drugs due to the absorption of molecules on the microchannels [50,51].

A considerable effort has been made to provide a hydrophilic character to the PDMS surface [52,53,54,55,56]. To improve microchannel wettability and overcome PDMS hydrophobicity, surface activation techniques are widely employed for PDMS surface oxidation. Additional methods used to address PDMS hydrophobicity include corona discharges, UV/ozone treatments, and oxygen plasma, which are frequently applied to PDMS surface oxidation to enhance microchannel wettability. The primary advantages of these technologies are their quick treatment times and ease of use; nevertheless, after a few minutes, contact with air causes the PDMS surface to regain its hydrophobicity [57,58,59]. However, Gökaltun et al. [60] have suggested an easy process that allows decreasing the hydrophobicity of PDMS for a prolonged amount of time without affecting its mechanical or transparent qualities. They have achieved this effect by employing copolymers made of polyethyleneglycol and PDMS segments (PDMS-PEG). He et al. [61] developed a PDMS surface coated with a layer of gold nanoparticles and PEG, using tannic acid as a reducing agent to enhance the properties of PDMS, making it resistant to biofilm formation and bacterial adhesion while also providing antibacterial effects through photothermal therapy (PTT). Lai and Chung [62] investigated the PEG coating on PDMS to improve hydrophilicity, demolding, and transparency in microfluidic chips. PEG1000 demonstrated better long-term hydrophilicity, higher transparency (55–70%), and a smooth surface after demolding. These findings position PEG1000 as ideal for rapid prototyping and optical observation with backlighting.

This review reports the most recent bioflow studies performed in PDMS devices at macro, micro, and nanoscale levels. Regarding the flow studies performed in microfluidic devices, this review focuses on the flow through contractions, bifurcations, and crossflow filters. Moreover, fabrication methods and PDMS biomodels obtained from medical images to perform hemodynamic studies are also revised. Therefore, the present review brings together the primary benefits, drawbacks, and difficulties associated with PDMS at various scale levels. Moreover, it can benefit researchers looking to improve their knowledge about this material and its applicability to perform blood flow studies, improve blood flow models, and validate numerical simulations.

## 2. PDMS Applications in Microfluidic Contractions

The most popular method to manufacture microfluidic devices is the photolithography. However, this microfabrication technique is expensive compared to the soft-lithography method [63,64]. In addition, there are alternatives that use cleanroom-less techniques to lower the cost of molds and microfluidic devices [65]. There are many different types of microfluidic devices, but one of the most important is to evaluate the behavior of pathological cells [66].

Blood cell deformability is a biomarker that may be used to distinguish between healthy and diseased cells [66]. Hence, PDMS microfluidic devices have been developed to enhance our knowledge and diagnose different diseases such as diabetes [67], malaria [68], cancer [69], and end-stage kidney disease [70]. In contrast to the blood rheology studies performed by rotational rheometers [71], cells flowing through PDMS microchannels have contractions. The deformability of the cells is affected by both shear and extensional flow, which represent the phenomena that happen in in vivo blood flow. Hence, due to the progress in microfabrication [65,72,73], microflow visualization [74,75,76,77,78], and image analysis techniques [77,78,79,80], several PDMS microfluidic devices containing abrupt and hyperbolic constrictions have been proposed to investigate the deformability of blood cells in conditions similar to in vivo microcirculation [66].

**Figure 1:**
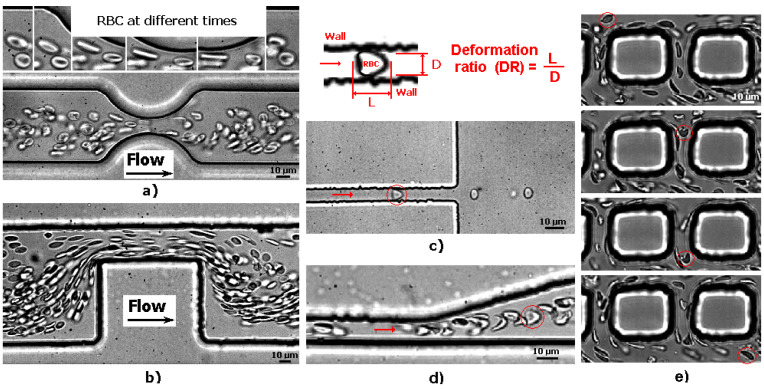
RBCs moving through a PDMS microchannel that has (**a**) a smooth and (**b**) an abrupt contraction, (**c**) rectangular PDMS microcapillary, (**d**) divergent region upstream of a rectangular PDMS microcapillary, and (**e**) micropillars (adapted from [66]).

To the best of our knowledge, one of the earliest PDMS microfluidic constriction channels to test blood cells’ deformability was performed by Shelby et al. [68]. They tested the deformability of malaria-infected red blood cells (RBCs) flowing through constriction microchannels, and it was found to be lower than that of healthy RBCs. After this deformability research assessment, many other PDMS microfluidic devices with constriction microchannels were developed to investigate the flow behavior and deformability of RBCs [81,82,83,84], white blood cells (WBCs) [85,86,87], and cancer cells [69,88]. These microfluidic devices fall into one of two categories: structure-induced deformation microchannel (the microchannel has a comparable or smaller dimension than the tested cells) or fluid-induced deformation microchannel (the microchannel is greater than the tested cells) [66,73]. Figure 1 shows examples of healthy RBCs flowing and deforming in microfluidic devices with fluid-induced and structure-induced deformation microchannels. It is clearly observed that the geometry of the microchannel affects how the RBCs deform.

The RBCs in microfluidic devices with structure-induced deformation microchannels are mostly deformed as a result of the walls’ high shear effects. Although these types of microdevices are extremely popular, they have several drawbacks due to the tiny dimensions of the microchannels (complexity of production, flow control, and visualizations of microflows). A potential solution to these challenges is to make use of the fluid-induced deformation microchannels. This approach is easier to fabricate and has the influence of both shear and extensional flows. They can be classified as microfluidic devices with abrupt, sudden, and hyperbolic constrictions [66,70]. An example of a PDMS device with abrupt or sudden microcontraction is the work performed by Zhao et al. [81], who assessed the RBCs deformation for different flow rates in a PDMS microchannel with a sudden contraction. According to their findings, the RBC elongation tends to reach a maximum value, and after that, the RBC stops deforming. More examples of PDMS devices with abrupt or sudden microcontractions can be found in the review by Lima et al. [66,70].

**Figure 2:**
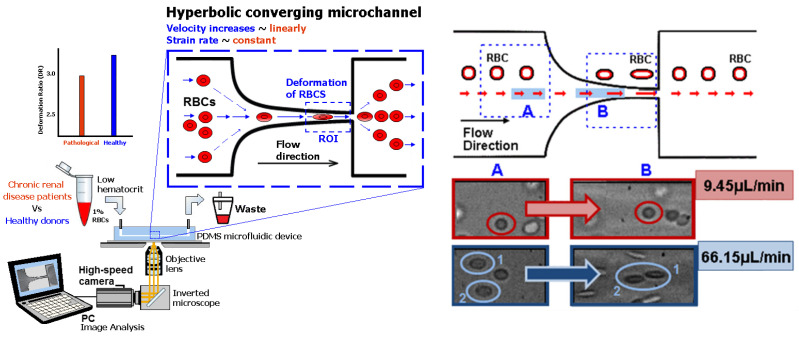
Deformation assessment in hyperbolic PDMS microchannels of healthy RBCs and RBCs of end-stage kidney disease patients. RBC deformability in a hyperbolic converging microchannel at two different locations (A) and (B) and flow rates (9.45 µL/min and 66.15 µL/min) (adapted from [66,70]).

Lima et al. [66] applied this finding to perform partial cell separation and deformability assessment in one step. This work assessed RBC deformability at both smooth and abrupt constriction microchannels (see Figure 1). More recently, Lima et al. [89] developed a cheap and easy-to-use particulate blood analog. They compared the deformability of both RBCs and micelles by a sudden-contraction microchannel. However, devices with sudden constriction do not provide uniform extensional flows and, as a result, many PDMS microfluidic devices with hyperbolic constrictions were used to determine the deformation of both healthy [83,90] and pathological blood cells [70]. Hyperbolic constrictions can generate homogeneous extensional flow. Therefore, it is possible to have a region with a constant strain rate [91]. Figure 2 shows the deformation behavior of RBCs flowing through hyperbolic PDMS microconstrictions at two different locations and flow rates.

**Figure 3:**
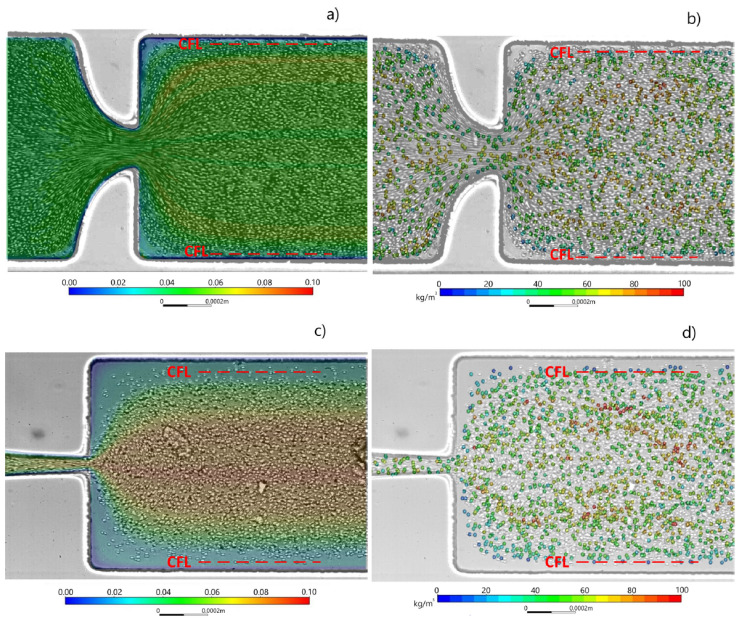
Simulated volume fraction distribution of RBCs in two different kinds of hyperbolic microchannels. Models applied: (**a**) Euler–Euler, (**b**) Euler–Lagrange, (**c**) Euler–Euler, (**d**) Euler–Lagrange. The experimental data are presented as a background image, and the simulated RBC volume fraction is presented as a color map [100].

As mentioned, Zhao et al. [81] and others [66] reported that in sudden contraction microchannels, the RBCs elongation achieves its maximal value, and they stop deforming any further. However, Zeng and Ristenpart [92] reported that the RBCs do not deform as they advance through the constriction of the microchannel. These conflicting results show the need for gaining further insights into the blood rheological phenomena at a micro-scale level, including cell-free layer (CFL), RBC motion, neighborhood interaction, orientation, and deformability. Hence, besides the experimental data, it is also crucial to develop and improve existing numerical flow models [93,94,95,96,97,98,99] in order to improve our understanding of the flow behavior in microvessels and microchannels. For instance, Gracka et al. [100] developed a multiphase numerical model with hybrid Euler–Lagrange and Euler–Euler techniques. They successfully validated this model by comparing their numerical simulations with the CFL formation downstream of hyperbolic contractions obtained from experimental data. Figure 3 shows the simulated RBC volume fraction distribution and comparison with the experimental flow images. These results show the importance of validating multiscale numerical models. Once validated, the numerical simulations can optimize the design, reduce the fabrication costs of microfluidic devices, and obtain more insights into the blood rheological properties at a micro-scale level, including the CFL formation and RBC deformability.

Fluids known as blood analogs are frequently employed in hemodynamic research as real blood presents safety concerns. Initially, glycerol and water combinations or xanthan gum diluted in glycerine and/or water were used as simple blood analogs [19]. However, these simple blood analogs do not allow for the research of different kinds of flow phenomena at the micro-scale level, including cell margination, plasma skimming, and the cell-free layer [65]. These well-known in vivo microscale phenomena do not happen when blood analogs do not have microparticles with dimensions close to blood cells. As a result, multiple studies have been conducted recently to create various types of particulate blood analog fluids for biomedical applications. These studies include changes in stiffness, shape, and size [89,101,102,103,104,105,106,107].

Pinho et al. [107] have suggested the use of particle blood substitutes comprising polymethylmethacrylate (PMMA) suspended in a liquid carrier composed of Dextran 40 and xanthan gum. In contrast with the works performed with simple blood analogs, they obtained cell-free layers downstream of microchannel contractions. The details about solutions, geometries, and results can be found in those references or a recent review by Sadek et al. [19]. However, these blood analogs using rigid microparticles have an extremely high probability of blocking microchannels. Therefore, these analogs do not replicate microscale blood flow phenomena. One way to overcome this limitation is by using flexible microparticles.

Zhang et al. [108] manufactured PFOB/PDMS-TPE core-shell microparticles using the porous glass membrane emulsification technique (SPG ME) with high production yields. The particles took on a concave shape without the need for additional deformation processes. By varying the pore size of the membrane and the composition of the dispersed phase, it was possible to precisely control the size and shape of the microparticles, which resemble human erythrocytes. They also showed high deformability and oxygen transport capacity, making them promising microcarriers for biomedical applications, such as tissue engineering.

In order to overcome these constraints, Choi et al. [109] and Lopez et al. [110] developed an easy emulsification technique to increase the production rate of the PDMS microparticles. The production of PDMS microparticles in square section microchannels using the blasting regime has been reported by Carneiro et al. [111]. The particles have diameters ranging from 27 to 59 μm, a maximum coefficient of variation of 17%, and a high droplet generation frequency (1.3 kHz), which enables the generation of thousands of microparticles per second. The microparticles are appropriate for performing microflow visualizations since they are free of impurities. However, parallelization is needed to increase the amount of generated PDMS microparticles. Another method proposed by Chen et al. [112] employed a 3D nested capillary microfluidic device to fabricate a large number of monodisperse PDMS microcapsules that can flow and deform like individual RBCs. However, this study did not show how they behave when they flow with a high concentration of PDMS microcapsules.

In summary, research and development are still in their infancy regarding blood analogue fluids that incorporate PDMS microparticles to simulate the behavior of blood cells. The production of monodisperse PDMS microparticles in large quantities, as well as the rigidity, aggregation, and agglomeration of these particles in the microchannels, are the most important problems that need to be solved for application in complex geometries such as bifurcations, constrictions, and crossflow microfluidic filters. These complex microchannels, including bifurcations, can also be manufactured with PDMS, and this is the main object of analysis in the following section.

## 3. PDMS Applications in Microfluidic Bifurcations and Complex Geometries

One of the main advantages of PDMS is its ability to cultivate endothelial cells on the surface of microchannels with circular geometries [113,114] and in rectangular cross-section microchannels with complex geometries such as microvascular networks [115,116,117,118]. This ability has promoted its use to make organs-on-a-chip platforms [119]: vascular-on-a-chip [120,121], heart-on-a-chip [122], kidney-on-a-chip [123], and lung-on-a-chip [124]. Moreover, PDMS combination with other materials [125], such as PMMA, allowed organs-on-a-chip diversification: liver-on-a-chip [126] and heart-on-a-chip [127].

As mentioned, PDMS properties extend to gas permeability, submicron structure replication, and transparency [128,129]. These unique properties allow it to be used to manufacture extremely complex geometries and devices at both macro and micro scale levels. Some successful examples are the production of microneedles for drug delivery and microfluidic systems [130], complex microchannel networks to mimic microvessels [131,132,133,134,135], and microfluidic devices to perform separation and sorting of blood cells [65]. Another astonishing PDMS application happened during the COVID-19 period, where PDMS was used to fabricate transparent face masks [136].

Geometries known as bifurcations and confluences can be found in sophisticated PDMS microfluidic devices, including organ-on-a-chip systems. Therefore, it is crucial to gain insights regarding the effect of these complex microgeometries on blood flow behavior. At the microscale level, the blood cells flow through bifurcation channels depends on a number of variables, including the size and dispersion of cells at the parent channel [137,138], the hematocrit distribution [138,139,140,141,142], the deformability and aggregation of cells [143,144,145,146].

**Figure 4:**
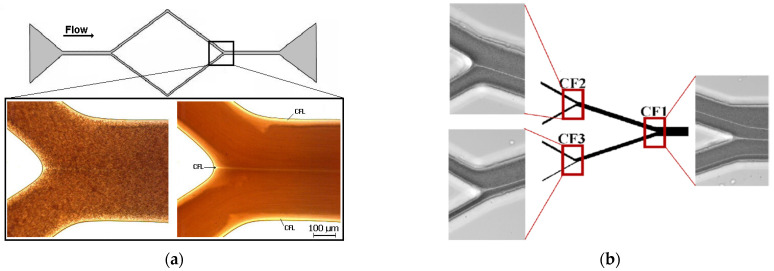
Micro blood flow visualizations of the CFL in (**a**) a simple PDMS microchannel geometry (manufactured by xurography) and complex in (**b**) a PDMS microchannel network (fabricated by soft lithography) composed of several divergent and convergent bifurcations. Adapted from [150,151].

Generally, blood flow in microfluidic devices displays distinct rheological behaviors and flow structures, including a high cell concentration in the core region and a CFL on the walls. [147]. However, earlier studies performed in PDMS microfluidic platforms manufactured by soft-lithography [148] and by xurography [149] have shown CFL at the walls and the confluence apex region. Later, Bento et al. [150] investigated the CFL formation in PDMS microfluidic devices with complex microchannel networks. These latter works have shown that hematocrit significantly affects the CFL. Moreover, microchannel networks with several convergent and divergent bifurcations are most likely to have CFLs on the walls and immediately downstream of the confluence apex in the middle section of the channels (see Figure 4). This study clearly shows that for hematocrits up to 15%, this flow phenomenon happens in PDMS microfluidic devices with microchannel networks and rectangular cross-sections. Further research is needed on whether this phenomenon also happens in circular microchannels and in vivo microvessels.

The separation of microparticles and blood cells based on their size, shape, deformability, and density is another potential use for PDMS microfluidic devices [65,152,153]. This capability can be extremely valuable since it can be applied to the diagnosis of blood pathologies.

According to the manipulating forces, these devices can be classified as active or passive devices [65,154,155,156]. Passive devices are the most commonly used due to their simplicity and lower manufacturing costs. For instance, Huang et al. [157], Choi et al. [158], and Karimi et al. [159] applied sequential pillar filters to perform blood cell separation and assessment. However, clogging and jamming appear at the pillar region when this technique is applied [65,160].

**Figure 5:**
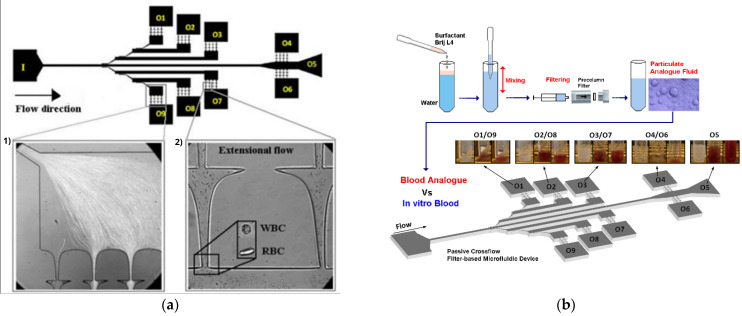
PDMS multi-step crossflow microfluidic device can separate and assess (**a**) healthy and pathological blood cells and (**b**) blood analog fluid produced by Brij L4 micelles and in vitro blood. Adapted from [163].

Therefore, several microfluidic devices, including crossflow filters, have been developed and optimized to minimize such problems [87,161,162,163]. Blood cells tend to travel tangentially along the pillars when the crossflow effect is used, as opposed to the conventional filtration procedures where the cells flow through the filter pillars and may cause clogging and jamming. To the best of our knowledge, Chen et al. [162] conducted one of the first studies to use PDMS crossflow pillars. In that work, they were able to separate the WBCs and RBCs from the blood plasma by successfully avoiding cell jamming by using layered filtering barriers [162]. Recently, Lima et al. further improved and optimized this system. They produced PDMS microfluidic devices that separate and assess healthy and pathological blood cells in one single step (see Figure 5a). Recently, this platform was used to investigate the capacity of a multi-step crossflow microfluidic device to separate a blood analog fluid produced by Brij L4 micelles [163] (Figure 5b).

## 4. PDMS Applications of Biomodels for Hemodynamic Studies

Biomodeling is a technique that involves the creation of physical models from biological representations and has been employed using Additive Manufacturing (AM) technologies. Over time, biomodels have evolved considerably, acquiring characteristics of flexibility, hollowness, and a more faithful representation of anatomy [164,165,166]. Currently, these models play a crucial role in optimizing surgical procedures, substantially contributing to reducing surgery time and mitigating the risks involved. However, in recent years, biomodels have assumed a preponderant role in studies of a hemodynamic nature. In this context, they are a highly relevant tool as they provide an effective approach for controlling experimental variables, as well as for validating and complementing numerical investigations [167,168].

For these biomodels to be suitable for hemodynamic studies, they must adhere to the following:

- Replicate the dimensions and reproduce the surfaces of real arteries.
- The lumen material must be easily removed during the manufacturing process and must not interact with the transparent material used.
- The biomodel material must be transparent and have the same refractive index as the experimental fluid.
- The manufacturing process must allow the construction of biomodels without dimensional discrepancies.

### 4.1. Manufacturing Process of PDMS Biomodel

Initially, the materials used to manufacture biomodels were glass [169,170], latex [171], and polymethyl methacrylate (PMMA) [172,173], but the rigidity, fabrication complexity, and high costs have reduced the interest of these materials to produce biomodels. Thus, PDMS has recently become the most used material [41] due to its good characteristics, such as transparency and flexibility, for faithfully replicating the arteries through its casting, which presents a very high resolution, reaching 6 µm × 6 µm [174,175].

Despite the advantages of using PDMS in biomodels, the manufacturing process is challenging. To replicate complex geometries with high fidelity and resolution, the Additive Manufacturing (AM) technique is the most used. However, it is still a challenge to print PDMS directly on a 3D printer due to the material’s curing process and viscosity, making it necessary to use a combined manufacturing process, which in this case is AM with PDMS casting.

**Figure 6:**
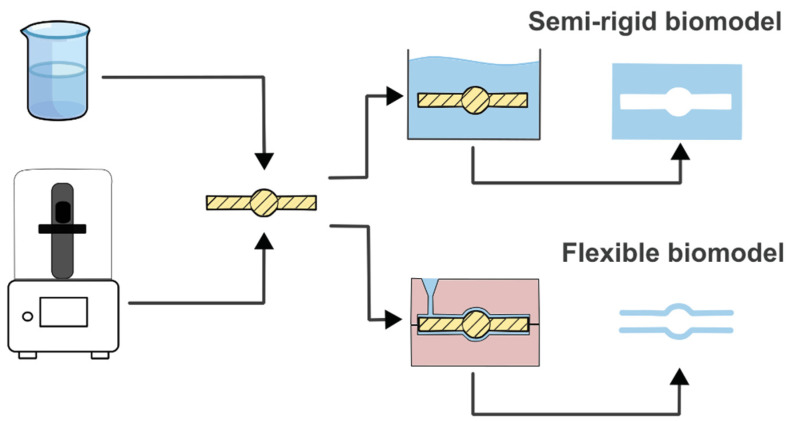
Manufacturing process of PDMS biomodels.

Figure 6 shows the process that has been used in the manufacture of PDMS biomodels. After obtaining the stl models, the artery lumen is printed with the 3D printer. Then, this physical model is placed in a container to generate semi-rigid models or inside a counter mold to generate flexible models. It is worth noting that the printed material must be destroyed at the end of the process, thus making the biomodel (phantom) completely transparent.

### 4.2. Hemodynamic Studies with PDMS Biomodels

**Figure 7:**
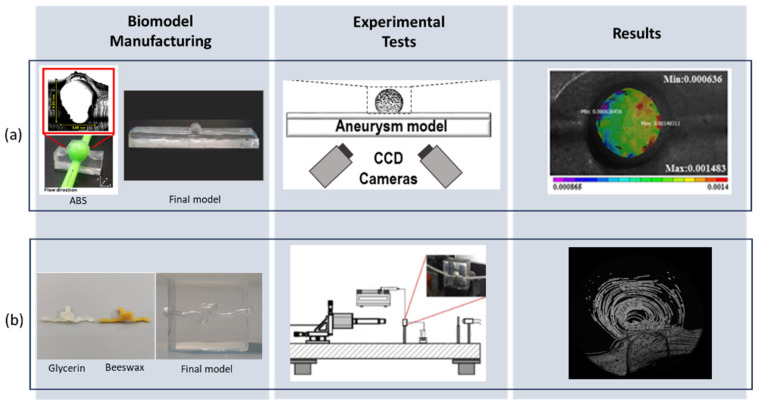
Experimental tests with biomodels: (**a**) Digital Image Correlation [177]; (**b**) video microscopy with particle tracking [30].

Some works listed the process of manufacturing biomodels of aneurysms, bifurcations, and stenoses. The intracranial aneurysm (IA) fabrication work used a combination of 3D printers and soft lithography techniques. In this work, the dimensions and geometry of the aneurysm model were based on clinical data for a common intracranial aneurysm [176], this being a simplified model previously designed in CAD software (Solidworks). To manufacture the biomodel, an ABS (Acrylonitrile Butadiene Styrene) mold was developed and printed on a printer 3. The PDMS was mixed with the curing agent and poured into the cavities of this mold so that the aneurysmal sac remained flexible. This technique made it possible to study the deformation of the wall for different flow rates using Digital Image Correlation (see Figure 7) [177].

One of the difficulties in manufacturing PDMS biomodels is finding materials that do not compromise the transparency of the PDMS and that are low-cost. Falk et al. [178] implemented and tested polyvinyl alcohol (PVA) to develop cheap and specific models made of the lost core casting technique; the models were measured and compared to the geometry in stl format, and the differences were practically insignificant and with visualization tests of particles, demonstrated that the model is suitable for experiments using the PIV technique. Following the same previous criteria of finding materials that do not compromise transparency and are accessible, the work of Souza et al. [30] demonstrated the manufacturing process of IA biomodels, in which an SLA printer was used to manufacture the lumen with high resolution in resin combined with a lost core molding technique (from paraffin, beeswax and glycerin soap). To validate the materials used, tests were carried out to analyze the dimensions of the biomodels in relation to the STL model and particle visualization tests. With this, they concluded that the biomodels made with beeswax and glycerin soap showed high transparency and good reproducibility, making them suitable for different experimental flow tests. Recent work by Sandy Karam et al. [179] developed an intracranial aneurysm geometry with the aid of a DLP printer and a resin that dilutes in water. The patient-specific IA biomodel was scanned using a Micro-CT to evaluate the geometry and dimensions and finally tested for transparency. They reached the conclusion that the produced samples could be utilized in in vitro research since they could successfully reproduce compatible and optically transparent aneurysms.

PIV is a modality frequently used to study flow in vitro, both for aneurysms and for coronary arteries and stenoses. PIV is an experimental method that measures the velocity field inside the region of interest by tracking the displacement of trace particles over time using high-speed cameras. The study by Ford et al. [180], for more direct validation, compared the detailed and predicted computational fluid dynamics (CFD) velocity fields with those measured using PIV. The tests were carried out on two anatomically realistic flow phantom biomodels; one was a giant aneurysm of the internal carotid artery, and the other was an aneurysm of the tip of the basilar artery, and both were constructed with a transparent silicone elastomer. The study demonstrated a good general agreement between PIV and CFD, showing the effectiveness of PIV in validating numerical models. The technique was also used to study risk factors related to aneurysm geometries. Li et al. [36] used PIV to measure the velocity of a blood-like fluid and were able to predict the effects of flow diversion caused by stent treatment. The study compared flow behavior with predictions from a CFD model. In their study, Brindise et al. [32] conducted one of the initial investigations on pulsatile volumetric particle velocimetry. They used two aneurysm models that were tailored to individual patients. This study employed a novel approach by using in vivo measurements, in vitro investigations, and in silico modeling. Specifically, in vivo 4D flow MR was used as boundary conditions for CFD and particle velocimetry.

Doutel et al. developed a method for manufacturing PDMS biomodels through rapid prototyping using lost core casting and sucrose. Models produced with sucrose casting allowed for excellent optical access for flow visualization and velocity field measurement using Micro-Particle Image Velocimetry (uPIV) [31]. In another study [18], employing the aforementioned process and a methodology for creating irregular stenoses [181], researchers examined the effects of geometry on in vitro and in silico blood flow. The researchers conducted a comparison between idealized models and patient-specific models of a coronary artery that was free from any disease. Additionally, they examined two derived models, one planar and one non-planar. The study found that when considering the individual patient’s anatomy, there was an uneven narrowing of the blood vessels, whereas in ideal scenarios, the narrowing was uniform and balanced. The importance of using patient-specific models in hemodynamic studies whenever possible was emphasized. The significance of eccentricity was also highlighted as a substantial parameter, as alterations along arterial branches resulted in asymmetric flow patterns. Moreover, the research findings indicate that non-planarity does not affect the development of stenosis, but it does have a significant influence on helicity. When the flow direction was altered to match the curvatures caused by non-planarity, there was a noticeable rise in helical flow.

In a research led by Jewkes et al. [182], 3D models of a healthy and stenotic porcine coronary artery were manufactured based on morphometric measurements. The study used two distinct printers to produce the resin and PDMS models (initially printed in wax). These models were then compared based on several criteria, including layer thickness, anatomical accuracy, and model production time. While resin may offer greater anatomical precision, the PDMS technique demonstrated superiority for functional testing objectives. During hemodynamic experiments using PDMS models, researchers detected the existence of helical flow patterns in the healthy models and identified the presence of recirculation zones in all biomodels. It is important to mention that these regions of recirculation were more noticeable in the models with stenosis. Although this study offers vital insights into the manufacturing process, there are various limitations, including the use of water as the working fluid and the recording of flow measurements using a cell phone. These aspects may compromise and diminish the accuracy of the results.

Interesting studies on stenotic bifurcation carotid arteries were conducted by Kefayati et al. [183,184,185], combining Particle Image Velocimetry (PIV) analysis with CFD simulations. Initially, researchers crafted life-sized flow phantoms of the carotid artery using PDMS (Sylgard^®^ 184, Dow Corning Canada, Inc., Calgary, AB, Canada; refractive index 1.41–1.43) through a lost-core casting technique. Three different artery configurations were considered for the study: a healthy carotid artery (normal, disease-free model) and 50% and 70% stenotic arteries [183]. The primary objective of the first study was to investigate transitional flows in the three fabricated models using PIV and Orthogonal Decomposition (POD), identifying the transition to complex flow in stenotic conditions. In another study [184], the authors explored not only the severity of stenosis (30%, 50%, and 70%) but also plaque eccentricity and ulceration, noting an increase in turbulence with greater stenosis severity and significant effects of eccentricity and ulceration. In a subsequent work [185], the authors analyzed the effects of these features (severity, eccentricity, and ulceration) on shear stress levels, highlighting increases associated with stenosis severity and notable differences between concentric and eccentric plaques. These three studies suggest that, beyond the severity of stenosis, other parameters may influence the risk of stroke, presenting serious clinical implications.

Choi et al. [186] conducted a significant in vitro study comparing constrictions in the neck arteries, both rigid and flexible, using a PIV method. They crafted a flexible constriction model through 3D printing, replicating real conditions of pulsatile blood flow. To simulate the thin fibrous layer, they carefully adjusted the composition of PDMS, controlling proportions, temperature, and curing time. While studying the effect of stenotic deformation on the pulsatile waveform and pressure drop, researchers observed that the flexible constriction changes its shape in response to alterations in inflow. This shape change results in an increased jet velocity and, consequently, a higher production of turbulent kinetic energy (TKE) compared to rigid models. This occurrence resulted in a phase delay in the highest point position of the waveform linked to the decrease in pressure across the stenosis. The findings indicate that it is possible to use the pressure drop waveform as a means of identifying susceptible flexible constrictions.

## 5. PDMS Applications with Nanoparticles and Nanofluids

Nanoparticles (NPs) and nanofluids (NFs) are gaining increasing interest in the biomedical scientific community due to their astonishing features at the nanoscale level: superparamagnetism, high surface-to-volume ratio, biocompatibility, and low toxicity, among others [187]. Static flow conditions at conventional plates tend to promote NPs sedimentation, and the results may not represent the in vivo environment. In contrast, using PDMS microfluidic devices makes flow conditions more realistic. Therefore, this approach is the most appropriate and accurate way for evaluating NPs in vitro. Hence, PDMS microfluidic devices have been developed to test NPs’ haemocompatibility, transport, toxicity, accumulation, and performance for drug delivery [188,189].

PDMS microfluidic models of human microvessels are frequently used to investigate NPs margination [190,191,192], shear stress effect on NPs accumulation [193,194], and interactions between blood cells and NPs [195,196]. Organ-on-a-chip models have also been used to evaluate the NPs’ transport. Additional information about this subject can be found in Zhu et al.’s review [189].

NPs toxicity has been evaluated by employing small organisms in microfluidic systems: zebrafish [197,198], *C. elegans* [199], and fruit flies [200]. For instance, the changes in *C. elegans* body length and width or their gene expression in a PDMS device can be employed to evaluate silver NPs toxicity [199].

As mentioned, PDMS organ-on-a-chip platforms are frequently used to evaluate the NPs’ efficacy. Agarwal et al. [201] employed a PDMS-glass microfluidic device and formed a 3D vascularized tumor model to test the anticancer efficacy of free DOX and DOX encapsulated in NPs. Other examples can be found in two review papers (Zhu et al. [189] and Ahn et al. [202]). In summary, these studies have shown that PDMS microfluidic devices have great potential to mimic the in vivo environment of human organs. This possibility enables researchers to test and predict the safety and efficacy of therapeutic drug candidates prior to clinical tests. In addition, the continuous progress in this field will accelerate the clinical adoption of NPs.

**Figure 8:**
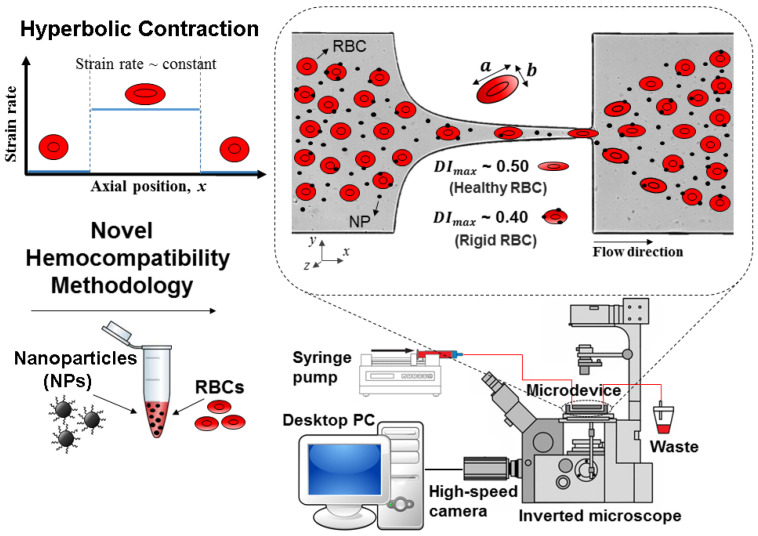
Representation of the procedure for evaluating the hemocompatibility of RBCs in contact with MNPs. The a and b represent the major and minor lengths of the ellipse, respectively. A high-speed video microscopy system and a PDMS microfluidic device with a hyperbolic constriction microchannel make up the microfluidic methodology, adapted from [208].

Most of the time, NPs for clinical applications are administered into the blood flow. As a result, it is crucial to test NPs haemocompatibility. Once the NPs are in contact with blood cells, they should not introduce any significant changes into the cells [203]. The conventional methods to evaluate NPs haemocompatibility involve the hemolysis analysis of RBCs [203,204], leukocyte phagocytosis and inflammation [205], platelet activation [206], and plasmatic coagulation test [207]. However, these methods have limitations and cannot assess multiple parameters such as transit time, recovery time, and cell deformability. Hence, Rodrigues et al. [208] developed the first PDMS high-sensitivity microfluidic device to identify small rigidity modifications of RBCs in the presence of NPs. They combined high-speed video microscopy with a hyperbolic constriction microchannel, and they were able to assess the impact of magnetic NPs on the human RBCs’ deformability. In contrast to other conventional hemocompatibility methods (such as the hemolysis analysis), they showed that a small number of NPs can affect the RBC rigidity. Furthermore, compared to conventional methods, this tool has demonstrated a higher sensitivity in detecting slight mechanical changes in the deformability of RBCs. Figure 8 shows a schematic diagram of this method.

A study conducted by Rodrigues et al. [208] employed a method to assess how RBCs mechanically respond to magnetic iron oxide (Fe_3_O_4_) nanocarriers. The results have shown that the maximum deformability achieved by the RBCs in contact with magnetic iron oxide NPs was lower than healthy cells. This study is essential for further understanding how cell–NP interactions occur in RBC disorders, and, as a result, it will open new avenues for developing novel nanocarriers as drug delivery systems.

**Figure 9:**
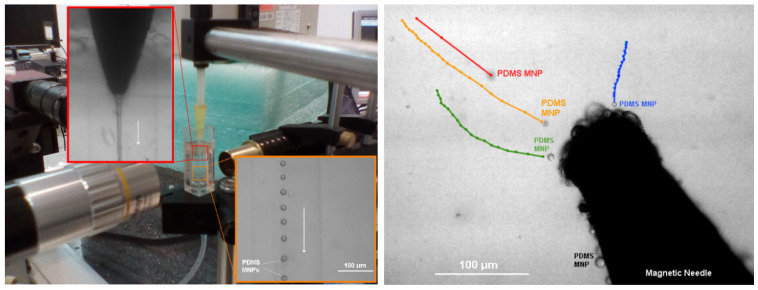
Experimental set-up and trajectories of magnetic PDMS microparticles produced by means of a flow-focusing technique, adapted from [209].

Nanotechnology progress has resulted in the development of a novel type of heat transfer fluid called NFs. In general, this fluid consists of nanoparticles that are evenly distributed in a base fluid. Adding NPs to the base fluid is claimed to enhance the thermal properties of the NF. Consequently, it has become the subject of research for numerous applications, including drug delivery and hyperthermia. An innovative NF was developed by Lima et al. [209], employing a flow-focusing technique. The researchers created magnetic PDMS microparticles by combining magnetic iron oxide nanoparticles with the elastomer pre-polymer. This NF has the potential to address critical issues related to the minimization of cluster formation and enhance the stability of the NFs. Figure 9 illustrates numerous trajectories of magnetic PDMS microparticles, exhibiting an upward trend in velocity as the microparticles approach the magnetic needle.

## 6. Conclusions

During the last 20 years, the use of PDMS to develop devices at both macro and micro scale levels has become a popular material due to the advantages it offers over conventional materials, including low cost, excellent optical transparency, easy-to-manufacture, and the ability for portable point-of-care devices. In addition, a distinctive advantage of the PDMS is its permeability to gases and, in this way, the ability to culture cells in closed channels, a task impossible to achieve in glass and other polymeric channels. The most critical limitation of PDMS is its hydrophobic nature, which increases the flow resistance and may limit some bioflow transport phenomena applications. Hence, in order to overcome such limitations, simple, fast, and effective treatments have been proposed to modify the surface of the PDMS into hydrophilic.

Despite being in its early stages of development, organ-on-a-chip models will advance further and become the most suitable method for assessing multifunctional drug delivery nanoparticles in the near future. In this way, PDMS will play a crucial role by integrating microfluidics in nanoparticle drug delivery testing.

Recent works performed with PDMS hyperbolic-shaped microchannels have demonstrated that by using this approach, it was possible to detect small mechanical changes in the RBCs’ deformability, a task not possible by using conventional methods.

The successful use of PDMS microchannels to replicate the circulatory system shows promise as a suitable application for investigating cardiovascular disorders. The exceptional hyperelastic properties and transparency of PDMS make it the preferred material for these specific applications. However, the hydrophobic characteristic of PDMS can also be a limitation, both in terms of blood flow and when attempting to cultivate endothelial cell cultures on its surfaces. Moreover, PDMS is crucial in the context of medical implant applications, primarily because of its biocompatible and hydrophobic characteristics. These attributes facilitate the creation of antimicrobial coatings for implants, which is an essential criterion in implant development. PDMS can also facilitate the creation of smooth surfaces using microfabrication techniques, which helps implant osseointegration in the body.

In summary, PDMS continues to offer innumerous opportunities to make significant advancements and developments in biomedical applications. As future work, it is important to continue investigating more methods to reduce the PDMS hydrophilic nature. Hence, it is crucial to create new techniques or enhance those that already exist in order to achieve a higher permanent hydrophilicity of PDMS. In this way, the formation of air microbubbles within PDMS microfluidic devices is more unlikely to appear.

## Figures and Tables

**Table 1:** Most relevant PDMS properties for microfluidic devices and in vitro biomodels.

Property (Unity)	Value	References
Refraction Index	1.4	[5]
Thermal conductivity (W/m∙K)	0.2−0.27	[6,7]
Young’s modulus (kPa)	360−870	[8]
Poisson ratio	0.5	[9]
Tensile strength (MPa)	2.24−6.7	[5,6]
Hardness (Shore A)	41−43	[10,11]
Hydrophobicity/contact angle (°)	~108° ± 7°	[12]
Melting Point (°C)	−49.9 to −40	[13]

**Table 2:** Main advantages and disadvantages of the most common materials used in microfluidic devices and biomodels [42,43,44,45,46].

	Main Advantages	Main Disadvantages
**PDMS**	Simple and low-cost fabrication, gas permeability, biocompatibility, optical transparency, variable elasticity, cell culture, easy fabrication (soft lithography).	Can absorb hydrophobic molecules, hydrophobic nature (can be modified), swelling in organic solvents.
**PMMA**	Low-cost fabrication compared to glass and silicon, optical transparency.	Rigid, thermal degradation and thermal oxidative degradation in the presence of oxygen, permeability inability, can deform under high pressure.
**3D printing resins**	Simple and low-cost fabrication, variable mechanical properties, ability to create complex geometries.	Inadequate optical transparency, low gas permeability, surface roughness, limited material choices depending on printer technology.
**Glass**	Optical transparency, inert, excellent roughness, and chemical resistance.	Rigid, fragile, expensive, difficult to reproduce complex geometries.
